# The Near-Gapless *Penicillium fuscoglaucum* Genome Enables the Discovery of Lifestyle Features as an Emerging Post-Harvest Phytopathogen

**DOI:** 10.3390/jof10060430

**Published:** 2024-06-18

**Authors:** Dianiris Luciano-Rosario, Wayne M. Jurick, Christopher Gottschalk

**Affiliations:** 1 Food Quality Laboratory, USDA-ARS, Beltsville, MD 20705, USA; d.lucianorosario@usda.gov; 2 ORISE Postdoctoral Research Fellow, Oak Ridge, TN 37830, USA; 3 Appalachian Fruit Research Station, USDA-ARS, Kearneysville, WV 25430, USA; christopher.gottschalk@usda.gov

**Keywords:** *Penicillium* spp., genomic resources, secondary metabolites, phytopathogen

## Abstract

*Penicillium* spp. occupy many diverse biological niches that include plant pathogens, opportunistic human pathogens, saprophytes, indoor air contaminants, and those selected specifically for industrial applications to produce secondary metabolites and lifesaving antibiotics. Recent phylogenetic studies have established *Penicillium fuscoglaucum* as a synonym for *Penicillium commune,* which is an indoor air contaminant and toxin producer and can infect apple fruit during storage. During routine culturing on selective media in the lab, we obtained an isolate of *P. fuscoglaucum* Pf_T2 and sequenced its genome. The Pf_T2 genome is far superior to available genomic resources for the species. Our assembly exhibits a length of 35.1 Mb, a BUSCO score of 97.9% complete, and consists of five scaffolds/contigs representing the four expected chromosomes. It was determined that the Pf_T2 genome was colinear with a type specimen *P. fuscoglaucum* and contained a lineage-specific, intact cyclopiazonic acid (CPA) gene cluster. For comparison, a highly virulent postharvest apple pathogen, *P. expansum* strain TDL 12.1, was included and showed a similar growth pattern in culture to our Pf_T2 isolate but was far more aggressive in apple fruit than *P. fuscoglaucum.* The genome of Pf_T2 serves as a major improvement over existing resources, has superior annotation, and can inform forthcoming omics-based work and functional genetic studies to probe secondary metabolite production and disparities in aggressiveness during apple fruit decay.

## 1. Introduction

*Penicillium fuscoglaucum* (synonym *P. commune*) is a blue-green-colored fungus that has been reported as a common spoilage organism of cheese and stored meat [1]. However, a recent report identified *P. fuscoglaucum* infecting apple fruit during post-harvest storage [2]. Conversely, *Penicillium expansum* is a ubiquitous post-harvest pathogen that is highly virulent on apple fruit and causes decay on a global scale [3]. Both fungi produce an array of secondary metabolites (e.g., patulin, cyclopiazonic acid [CPA]) and have been found to cause the spoilage of cured meats and cheeses [1,3]. *P. expansum* produces patulin, a detrimental mycotoxin that can also be a threat to food safety [3]. On the other hand, *P. fuscoglaucum* can produce CPA, which has been shown to cause focal necrosis in vertebrates when present at high concentrations [4]. Recent phylogenetic analyses showed *P. fuscoglaucum* to be an ancestral species to the modern cheese-making *Penicillium* such as *P. biforme* and *P. camemberti* [5,6,7]. The *Fasciculata* clade is closely related to the *Penicillium* group that contains *P. expansum* and *P. digtatum* [8]. These phylogenetic relationships and the wide host range of *P. fuscoglaucum* makes it an interesting species to study to determine factors mediating host range and infection biology. However, to explore the genetic and molecular mechanisms underlying fungal lifestyle and biology, high-quality genomic resources are needed.

Ropars et al. (2015) [6] reported the first genome assembly of *P. fuscoglaucum* (FM041) that was generated using next-generation sequencing approaches. That assembly is considered scaffold-level but is highly fragmented, with the scaffold number being >900 and an N50 of 245 kb. Since the release of the first genome, three additional genomes of *P. fuscoglaucum* have been assembled and released (NCBI Bioproject PRJNA644637). However, the three later genomes did not offer a substantial improvement in genome quality. With the availability of third-generation sequencing technologies, achieving chromosomal-scale and nearly complete genome assemblies of fungi is possible [9,10]. Herein, we present the genome assembly and annotation of *P. fuscoglaucum* based on third-generation sequencing, resulting in a highly contiguous assembly.

Secondary metabolites or natural products are compounds produced by different species including plants, bacteria, and fungi [11]. These compounds have diverse ecological roles which include signaling molecules, toxins, and antimicrobial compounds, which have been mined to produce lifesaving drugs. In fungi, secondary metabolites are synthesized due to the products of biosynthetic gene clusters (BGCs). BGCs are arranged as genes in close proximity (i.e., clusters) that encode for the necessary gene products to synthesize a secondary metabolite. The size of BGCs can range from 2 to more than 20 genes [12]. Historically, natural product discovery has not only aided in the elucidation of food and animal safety threats such as the identification of aflatoxin and its relationship to Turkey X disease but also have been utilized for pharmacological purposes such as the large-scale production of penicillin. Since these and many other discoveries, mining fungal genomes for natural products has been of great interest to the scientific community and biotechnology industry. Therefore, there is a need to characterize and mine fungal organisms and their genomes to predict and identify potential beneficial secondary metabolites. Progressive efforts have been made to obtain comprehensive data on *P. expansum* secondary metabolites, their regulation, and potential roles [13,14,15,16,17,18]. In the case of *P. fuscoglaucum*, only a few studies have focused on expanding the current data on its secondary metabolism repertoire; thus, the genome we report presents great opportunities for secondary metabolite discovery [1,19,20].

The *P. fuscoglaucum* isolate we sequenced was cultured during another experiment focusing on the *Penicillium* spp. infection of apples under post-harvest storage conditions. The *P. fuscoglaucum* isolate was obtained on selective medium and displayed a reduced virulence phenotype in apple; thus, it was sampled and sequenced. The resulting genome assembly exhibited uncharacteristic synteny with other *P. expansum* genomes and was hypothesized to be another species. We set out to identify the resulting species using two independent methods. Based on the results of those efforts, we named our assembly Pf_T2 as *P. fuscoglaucum.* The source of *P. fuscoglaucum* isolate Pf_T2 remains unknown but is suspected to be an indoor airborne contaminate that was cultured on selective medium. Data from this work shows that *P. fuscoglaucum* can infect apple fruit, albeit at a lesser level compared to the more aggressive *P. expansum* Pe21 isolate. The genome is near gapless and will serve as the gold standard to facilitate omics-based, functional, and comparative studies in the fungus to explore molecular mechanisms that mediate host–pathogen interactions and the production of secondary metabolites. 

## 2. Materials and Methods

### 2.1. Isolate Origin and Culturing 

The *P. fuscoglaucum* Pf_T2 isolate was obtained during routine culturing in the Food Quality Laboratory in building 006 at the Agriculture Research Service Beltsville Research Center, Beltsville, MD on selective medium. Our goal was to screen transformants for single gene deletion events in the highly virulent *Penicillium expansum* post-harvest apple fruit pathogen. However, during this process, one isolate with different morphology (color) was obtained, most likely as an indoor air contaminant. The isolate was single-spore purified and cultured on Glucose Minimal Medium (GMM) (10 g/L glucose, 1X Nitrate Salts, 1X Trace Elements, 16 g/L agar, pH 6.5) at 25 °C to record fungal growth in vitro. 

### 2.2. DNA Extraction

DNA was extracted as previously described [21]. Briefly, Pf_T2 conidia were incubated in 10 mL of Liquid Minimal Media and Yeast Extract (LMM) (10 g/L glucose, 5 g/L yeast extract, 1X Nitrate Salts, 1X Trace Elements, pH 6.5) for 36 h at 25 °C. The mycelial mat was collected and lyophilized until dry. Approximately 0.1 g of tissue was macerated in a 1.5 mL tube using a sterile toothpick until obtaining a fine powder. Then, 500 µL of LETS buffer (10 mM EDTA (pH 8), 0.5% SDS, 10 mM Tris-HCl (pH 8), 0.1 M LiCl) was added, and the mixture was shaken by inversion and incubated for 5 min at room temperature. After this, 500 µL of phenol–chloroform–isoamyl alcohol (PCI) (25:24:1) was added, mixed by inversion, and incubated at room temperature for 5 min. The mixture was centrifuged at 16,873× *g* for 10 min at 4 °C. The supernatant was transferred to a 1.5 mL tube, and 500 µL of PCI was added. The samples were centrifuged again at 16,873× *g* for 10 min at 4 °C. The supernatant was then transferred to a clean 1.5 mL tube, and 1 mL of 100% ethanol was added for DNA precipitation. The DNA was then pelleted by centrifugation using 16,873× *g* for 10 min at 4 °C. The supernatant was discarded, and the pellet was washed by adding 500 µL of 70% ethanol and then centrifuging the tubes at 16,873× *g* for 2 min at room temperature. After this, the supernatant was discarded, and the DNA pellet was dried at room temperature. The DNA was then reconstituted in 10 mM Tris Buffer (pH8) (Invitrogen, Carlsbad, CA, USA), and RNAse treatment was performed by incubating the samples at 65 °C for 30 min to an RNAse final concentration of 10 µg/mL. The DNA was then visualized via agarose gel electrophoresis and ethidium bromide staining. The extracted High-Molecular-Weight (HMW) DNA underwent short-read elimination (SRE) to enrich for fragment lengths of >10 Kb using the XL SRE kit from Circulomics (Baltimore, MD, USA).

### 2.3. Oxford Nanopore Third Generation Sequencing

The HMW, size-selected DNA was evaluated for quantity and quality using a Qubit fluorometer (ThermoScientific, Waltham, MD, USA) with a high-sensitivity genomic DNA fluorescent kit and a TapeStation 4150 (ThermoScientific) using the genomic DNA screentape and ladder for fragments > 50 Kb. Following HMW DNA quality checks, Oxford Nanopore Technologies (ONT; Oxford, UK) sequencing libraries were prepared using a V14 kit with duplex reads (SQK-LSK114). The resulting libraries were checked for quality and quantity using the TapeStation as described previously. The ONT library was sequenced for ~72 h on an ONT R10.4.1 flow cell using a MinION device. Reads of an estimated < 1000 bp were filtered using the sequencing software MinKNOW (v23.04.6), real-time basecalling was disabled, and POD5 files were chosen to be the output. POD5 files were used as the input into the Dorado (v0.3.3; ONT) basecaller. The basecalling was performed using the super-accurate (SUP) basecalling model dna_r10.4.1_e8.2_400bps_sup@v4.2.0. The resulting fastq file was then checked for overall quality and quantity using NanoPlot (v1.40.0; De Coster and Rademakers 2023). Reads were processed for adapter sequences using Porechop (v0.2.4; https://github.com/rrwick/Porechop [accessed on 27 September 2023]) with default settings. The fastq was further filtered for only reads ≥20 kb using NanoFilt (v2.8.0) [22].

### 2.4. Fungal Genome Assembly and Annotation

The genome was assembled using Flye (v2.9.2; [23] with the --nano-hq option. We used an estimated genome size of 32.4 Mb and enabled the --scaffold option. The resulting assembly was analyzed using genometools seqstat (v.1.6.2; http://genometools.org/ [accessed on 17 September 2023]). Genome assembly completeness was assessed using BUSCO (v.5.3.1) [24] and the eurotiales_odb10 database. We used seqtk (v1.4; https://github.com/lh3/seqtk [accessed on 17 September 2023]) to filter sequencing artifacts and contigs/fragments with coverage > 200. The resulting filtered assembly was then annotated for repeats using RepeatModeler (v.2.0.1) [25] and RepeatMasker (v.4.1.1; [26] on the GenSAS server (v6.0)) [27]. A consensus repeat annotation GFF file was generated using One code to find them all (v1; https://doua.prabi.fr/software/one-code-to-find-them-all [accessed on 27 September 2023]) on the GenSAS server. Gene annotation was performed using MAKER2 (v.3.01.03) [28] with protein evidence from the *P. camemberti* genome obtained from NCBI (GCA_000513335.1) [29] as the input. We performed ab initio gene prediction within MAKER2 using two rounds of training for Augustus (v.3.4.0) [30] and SNAP (v2006-07-28; Korf 2004). We used BLASTP (v2.14.0) [31] and Interproscan (v5.61-90.3) [32] to assign functional annotation to the MAKER2 annotation. Annotation statistics and genome quality were assessed using AGAT (v0.9.1; https://github.com/NBISweden/AGAT#publication-using-agat [accessed on 1 May 2022]) and BUSCO. The non-coding genes (rRNA and tRNA) were then annotated using the Infernal software (v1.1.4) [33]. 

### 2.5. Species Level Identification

The first method to identify the species of our genome was to construct a phylogenetic tree of 34 typed specimen reference genomes of the *Penicillium* spp. on NCBI using average nucleotide identity. We performed this using the fastANI program and an automated clustermap figure generation script ANIclustermap [34]. Our second approach used the BUSCO genes from the eurotiales_odb10 database to reconstruct a phylogenetic tree (https://github.com/jamiemcg/BUSCO_phylogenomics?tab=readme-ov-file [accessed on 10 October 2023]). Here, we performed the phylogenetic tree construction using a subset of 19 genomes implemented during the fastANI analysis. Lastly, we performed a whole genome sequence alignment between our Pf_T2 assembly and the NCBI reference genome FM041 for *P. fuscoglaucum* (GCA_000576735.1) [6]. This alignment was performed using the standalone version of D-Genies using minimap2 as the aligner and the parameter “many repeats” enabled [35].

### 2.6. Radial Growth Assay and Apple Fruit Virulence Assays

Spore suspensions for *P. expansum* and *P. fuscoglaucum* were obtained from 7 d old cultures grown on GMM plates at 25 °C. The spore suspensions were then diluted to a final concentration of 10^6^ spores/mL. For the radial growth assay, GMM plates were inoculated with 10 µL of the diluted spore suspension, and the colony radial growth was measured for 7 d after inoculation. Three replicates were assessed. For the apple fruit virulence assay, organic Honeycrisp apples were washed, sprayed with 70% ethanol, and dried using paper towel. Then, the apples were wounded and inoculated with 10 µL of the diluted spore suspension. The lesion diameter was measured for 7 d after inoculation. Five replicates were assessed.

## 3. Results

### 3.1. Penicillium fuscoglaucum Pf_T2 Genome Sequencing

The ONT sequencing yielded 1.7 M reads and a total of 15.3 Gbps. The N50 length was 14.3 Kb with a mean read quality score of 14.9. Following the filtering of the raw reads, 5.3 Gbp of sequence was retained with a mean read length of 31.5 kb. This corresponds to an estimated coverage of >160×, which is sufficient for accurate de novo genome assembly. Using the filtered long reads, Flye generated an assembly of 20 contigs and a total length of 35.1 Mb. The N50 of the contigs was 9.1 Mb, suggesting a highly contiguous assembly (Table 1). The further inspection of the contigs found that 15 of the 20 were relatively short (<5000 Kb) and exhibited a coverage >160×. These descriptive genome statistics suggested that the short contigs were sequencing artifacts or were of plastid origin. As a result, these contigs were removed from the assembly. Overall, the current assembly is the most contiguous genome currently available for *P. fuscoglaucum* [6] (Table 1). 

### 3.2. Pf_T2 Genome Annotation

Following species-level identification, we conducted genome annotation for repetitive elements, coding, and noncoding genes. RepeatMasker and RepeatModeler identified 10,784 low-complexity and interspersed repeats and 3814 de novo repeats, respectively (Table 2). In total, 14,598 consensus repeats were identified and subsequently masked for gene annotation. Due to a lack of RNA-seq data both in-house and in public databases, we undertook a protein homology-based approach for the identification and annotation of genes. The MAKER2 annotation pipeline predicted a total of 11,616 genes. The average number of exons per mRNA was 3.1, with an average mRNA length of 1658 bp. The gene count is within the anticipated range of 9000 to 14,000 for other recently published *Penicillium* spp. [10,36,37]. A BUSCO analysis of the proteins encoded by the annotated genes found the genome to be 97.9% complete. The analysis found 4102 complete BUSCOs compared to only 38 fragmented and 51 missing of the 4191 total BUSCOs in the eurotiales_odb10 database. The infernal pipeline further annotated a total of 267 noncoding genes, of which 147 were annotated as tRNAs. Furthermore, tRNAscan-SE identified another 717 tRNA loci (Table 2). These statistics illustrate the thorough annotation of our high-quality genome.

### 3.3. Penicillium Phylogenomic Analysis Reveals Isolate Sequence to Be P. fuscoglaucum

**Figure 1:**
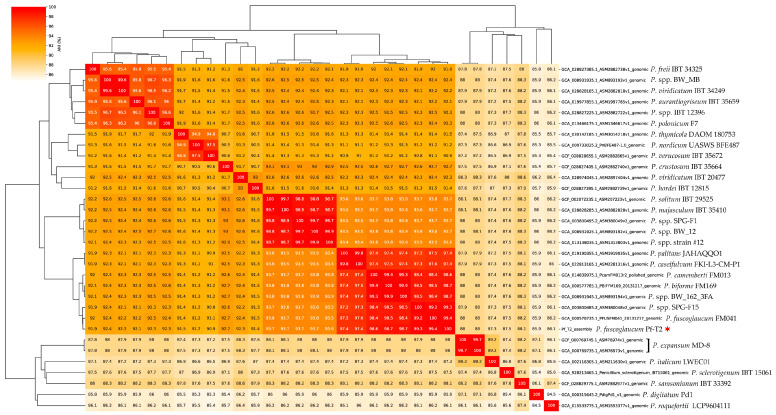
FastANI phylogenetic tree and heatmap of different *Penicillium* type specimen reference genomes, including the Pf_T2 genome marked by a red colored star.

**Figure 2:**
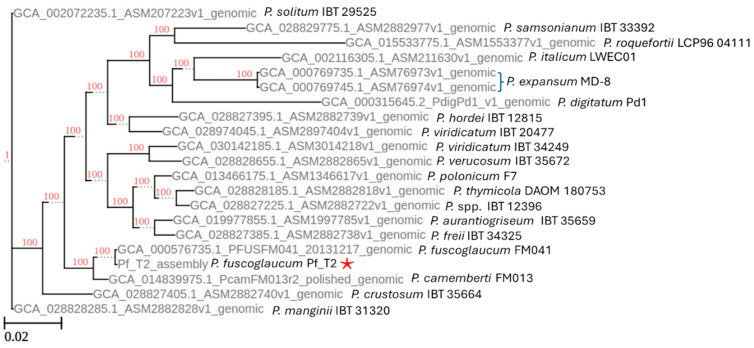
BUSCO phylogeny of nineteen different *Penicillium* type specimen reference genomes with the *P. fuscoglaucum* Pf_T2 genome marked by a red star. Each genome is also indicated with corresponding genus and species name followed by isolate name.

**Figure 3:**
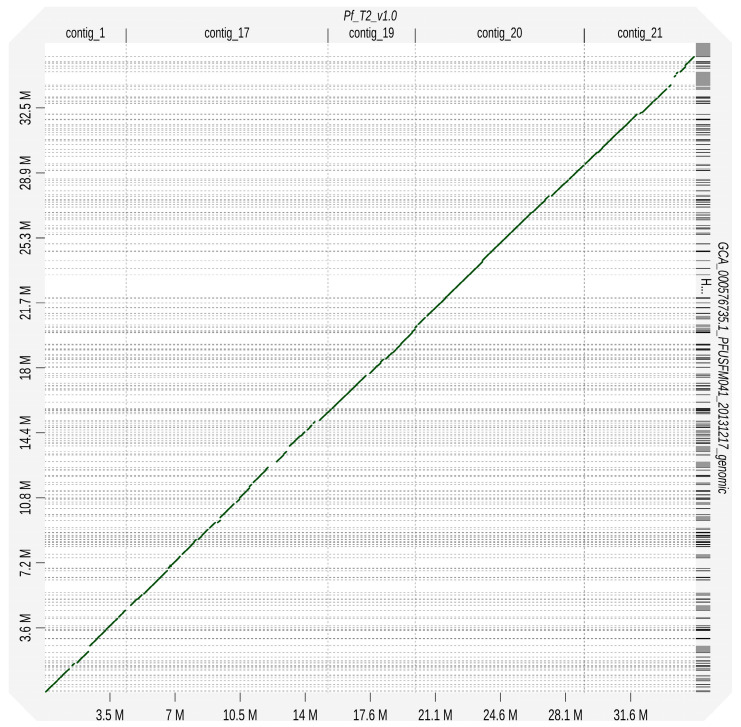
Collinearity with whole genome alignments between Pf_T2 assembly and the NCBI reference genome FM041 for *P. fuscoglaucum*.

Due to the lack of precise origins of this isolate and the reduced virulence phenotype in apple fruits, we employed an orthogonal approach. First, we utilized an average nucleotide identity (ANI) method to compare against all NCBI reference genomes within the *Penicillium* genus. The ANI analysis identified a close grouping between our genome *P. camemberti*, *P. roqueforti*, and other *Penicillium* spp. associated with blue cheese production. We reperformed the analysis with a narrower subset of assemblies that included the closest relatives from the first analysis but included *P. expansum* and its nearest relatives as an outgroup (Figure 1). These results indicated that our genome was closest to *P. fuscoglaucum*. Our second approach utilized the BUSCO genes of the eurotiales_odb10 database. Here, a subset of reference genomes was analyzed through the pipeline, including the reference *P. cambeterti* and *P. fuscoglaucum.* Similar to the fastANI results, our genome formed a distinct clade with *P. cambeterti* and *P. fuscoglaucum* (Figure 2). Our assembly was grouped closest to the reference *P. fuscoglaucum* FM041 assembly. When comparing whole genome alignment between the NCBI reference *P. fuscoglaucum* FM041, we observed striking collinearity (Figure 3). These multiple independent lines of evidence are confirmation that our Pf_T2 genome is *P. fuscoglaucum*. Moreover, our assembly vastly improves upon the available genomic resources for this species.

### 3.4. P. fuscoglaucum Can Cause Rot in Apple Fruits and Is Less Virulent When Compared to P. expansum

**Figure 4:**
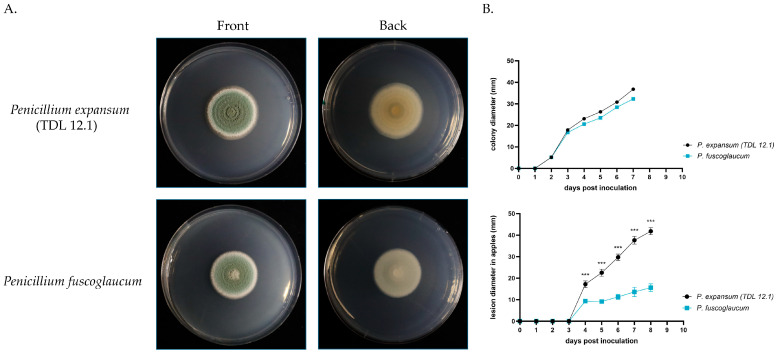
*P. fuscoglaucum* Pf_T2 isolate and *P. expansum* TDL 12.1 growth and virulence. (**A**) Growing in culture on glucose minimal medium. (**B**) Lesion diameter in “Honeycrisp” apple fruit over time. Error bars denote standard deviation of the mean. Asterisks denote significant differences between strains *** *p* < 0.001.

The cultural characteristics of the Pf_T2 fungal culture are congruent with what has been reported for *P. fuscoglaucom,* including dark green coloration [7]. Growth kinetics and colony diameter in vitro on GMM showed that the Pf_T2 isolate had very similar values over time to an aggressive apple pathogen *P. expansum* strain 12.1. (Figure 4). The colony diameters in these two species were not statistically significantly different after performing a t-test at every time point. However, when the *P. fuscoglaucum* Pf_T2 isolate was inoculated into wounded apple fruit, it resulted in approximately four-fold smaller lesion diameter (value) compared to the *P. expansum* apple fruit pathogen strain 12.1 (Figure 4). This difference is statistically significant 4 to 7 d post-inoculation after performing a *t*-test at every timepoint.

### 3.5. Secondary Metabolic Gene Cluster Identification

The annotated genome sequence from *P. fuscoglaucum* PF_T2 was analyzed using the AntiSMASH web program version 7.0 and yielded an array of partial and intact Secondary Metabolic (SM) gene clusters [38]. Emphasis was placed on clusters with identity of 70% or greater which showed eight different SM gene clusters that include YWA1, fumihopaside, cyclopianzonic acid, nidulanin A, andrastatin A, choline, PR-toxin, and burnettiene A (Table 3). Along with percent identity, contig number for each SM cluster location is indicated and allows for an in-depth analysis of these findings. We report this as the initial description of SM clusters for *P. fuscoglaucum,* as the presented genome is the most complete to date. 

## 4. Discussion

Genomic resources are the base component of fungal taxonomy, genetic diversity studies, genome mining for biotechnological applications, and functional genomics. By having high-quality genomic data, the resolution of intraspecies comparisons and evolutionary biology will increase. Here, we present various lines of evidence that identify an isolated strain and its high-quality genome as the understudied *P. fuscoglaucum*. In addition, we show its potential role as a post-harvest pathogen of apples while also presenting its decreased virulence when compared to *P. expansum*, the main causative agent of blue mold disease. Furthermore, we explore part of the potential secondary metabolome of the species.

In this study, we present a high-quality genome for *P. fuscoglaucum*. By using Oxford Nanopore Technologies, we acquired long-read sequences with a mean read length of 31.5 kb. After filtering and annotation, the genome size was 31.5 Mb, similar to the average 33.27 Mb of 93 recently sequenced *Penicillium* spp. [37] This high-quality assembly led to the ability to generate a final annotation that contained 11,616 genes, within the expected range for the species. In addition, we evaluated the virulence of this species on apples and compared it to *P. expansum*. The disparity in virulence between the two species in apple fruit but not in axenic growth, which affords an opportunity to use these two strains to investigate molecular mechanisms underpinning these observations, since it is difficult to generate single gene deletion strains that display significant defects in apple fruit decay while having very similar if not identical growth rates in culture [39]. Similar studies have used different *Penicillium* spp. displaying varying levels of virulence to use as practical models to investigate genes, pathways, and metabolites involved in fungal-mediated apple fruit decay [40,41]. Hence, this near-gapless *P. fuscoglaucum* Pf_T2 genome, with its high-quality assembly and annotation, will serve as a solid foundation for omics (transcriptomic, proteomic, metabolomic) and functional genetic studies (e.g., gene knockout) to investigate virulence mechanisms. It can also serve as a platform to identify pathways and gene clusters encoding proteins involved in the production of small molecules of interest in this unique species. 

Secondary metabolites or natural products are an invaluable source for biotechnological use. These are also important due to health concerns. When evaluating a subset of the potential secondary metabolome, we found that *P. fuscoglaucum* is predicted to encode eight clusters that share 70% identity to a previously identified SM cluster. Nevertheless, the presence of an unknown biosynthetic gene cluster (BGC) provides a discovery opportunity for the species. Of these identified clusters, most have been characterized to be produced in different species, e.g., fumihopaside in *Aspergillus fumigatus*, cyclopiazonic acid in many *Penicillium* spp., nidulanin A in *A. nidulans*, burnettiene A in *P. camberti*, and PR toxin in *P. roqueforti* [42,43,44,45,46]. In the same manner, most of these have been predicted to be present bioinformatically by various *Penicillium* spp., but the production of the compound is still to be determined. Still, these results indicate the presence of clusters may not be intact and do not indicate the definite production of the corresponding compound. 

**Figure 5:**
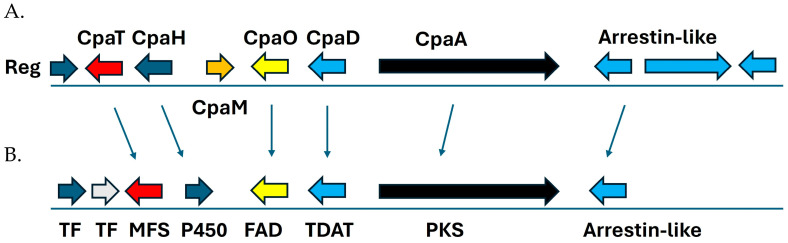
Cyclopiazonic acid (CPA) biosynthesis cluster in *Penicillium camemberti* species complex that includes *P. fuscoglaucum*. (**A**) CPA cluster in *P. camemberti* var. “*camemberti*” reference genome FM013 recreated from Ropars et al. 2020 [7]. The cluster contains 8 loci with 6 CpA genes: Reg (transcriptional regulator/C6 transcription factor), CpaT (multi-function substrate transporter), CpaM (hypothetical protein), CpaH (Cytochrome P450), CpaO (FAD oxidase), CpaD (dimethylallyl synthase), CpaA (Non-ribosomal polyketide synthase), and Arrestin-like gene. (**B**) CPA Cluster in *P. fuscoglaucum* isolate Pf_T2 isolate contains 7 of the 8 genes present in *P. camemberti* gene cluster and is missing cpaM (hypothetical protein) and contains an extra transcription factor. TF (transcriptional regulator C6 transcription factor), TF (Gal4 transcriptional regulator), MFS (multi-function substrate transporter), P450 (Cytochrome P450 monoxygenase), FAD (FAD oxidase), TDAT (Tryptophan Dimethylallyl transferase), PKS (polyketide synthase), and Arrestin-like protein.

Cyclopiazonic acid (CPA) is a mycotoxin and fungal neurotoxin that was originally isolated from *Penicillium cyclopium* and is produced by *P. griseofulvum*, *P. camemberti*, *P. commune*, *Aspergillus flavus*, and *A. versicolor* [44]. Recently *P. biforme* and *P. fuscoglaucum* were shown to contain six CPA genes (T, H, M, O, D, and A) and produced detectable amounts of cyclopianzonic acid (CPA) [7]. Each gene in the cluster encodes proteins/enzymes needed for CPA biosynthesis including: CpaT (multi-function substrate transporter), CpaM (hypothetical protein), CpaH (Cytochrome P450), CpaO (FAD oxidase), CpaD (dimethylallyl synthase), and CpaA (Non-ribosomal polyketide synthase). The analysis of the Pf_T2 genome revealed 5 of the 6 CpA genes (T, H, O, D, and A) along with the 5′ flanking regulator and 3′ arrestin-like locus (Figure 5). However, differences between the strain from Ropars et al. 2020 [7] and Pf_T2 includes the lack of CpAM, CpAH being transcribed in the opposite direction, and one extra transcription factor at the 5′ end of the cluster. A lack of CpAM encoding a hypothetical protein may be due to a recombination event in our strain and/or could be an artifact of gene annotation. We hypothesize that our strain will produce CPA, even with the apparent lack of the CpAM gene, since *Aspergillus* spp. only contains three loci: CpAS (PKS), D (Dimethylallyl transferase), and O (FAD oxidase) [47]. However, CPA production by *P. fuscoglaucum* Pf_T2 in vitro remains to be seen and is the subject of a future investigation in our lab.

## Figures and Tables

**Table 1:** Genome assembly statistics of *P. fuscoglaucum* genomes.

Genome/Strain Name	Pf-d2	FM041	FJII-L5-SW-P3	S/N-202-OC-P2	VS AB I KN 5
Total Length (Mb)	35.1	36.1	36.4	36.1	36.0
Scaffold/Contig Number	5	953	1188	1126	1126
N50 (Kb)	9100.0	245.0	203.9	146.0	145.9
GC Percent		47.5	47.5	47.5	47.5
BUSCO ^a^					
Publication	This study	Ropars et al., 2015 [6]	-	-	-
NCBI Genome Assembly		PFUSFM041_20131217	ASM2281336v1	ASM1980454v1	ASM1980455v1
NCBI Reference Genome	No	Yes	No	No	No
GenBank Assembly ID		GCA_000576735.1	GCA_022813365.1	GCA_019804545.1	GCA_019804555.1

^a^ BUSCO run using genome mode and Eurotiales ODB10 (*n* = 4191).

**Table 2:** Genome annotation statistics.

Repeat Annotation	Features
RepeatMasker	10,784
RepeatModeler	3814
Consensus	14,598
**Gene annotation**	**Value**
Protein Coding Genes	11,616
Total Exons	36,354
Average Exons per mRNA	3.1
Average mRNA Length (bp)	1658
Total Gene Length (Mb)	19.27
tRNA ^a^	717
tRNA ^b^	147
Noncoding Genes (non-tRNA)	120
BUSCO ^c^—Complete Genes	4102 (97.9%)
BUSCO ^c^—Fragmented Genes	38 (0.9%)
BUSCO ^c^—Missing Genes	51 (1.2%)

^a^ tRNAscan-SE; ^b^ Infernal; ^c^ BUSCO run using genome mode and Eurotiales ODB10 (*n* = 4191); percent of total is in parentheses.

**Table 3:** Secondary metabolite gene cluster prediction via AntiSMASH for *Penicillium fuscoglaucum* Pf_T2 isolate. Contig number, region, type, cluster, and % identity are indicated for those >70% identity.

Contig Number	Region	Type	Cluster	% Identity
1	n.d.	n.d.	n.d.	n.d.
17	2.2	Type 1 polyketide synthase	YWA1	100%
17	2.6	Terpene	Fumihopaside	100%
17	2.12	Non-ribosomal peptide synthetase, Type 1 polyketide synthase, idole	Cyclopiazonic acid	71%
17	2.21	Non-ribosomal peptide synthetase, Type 1 polyketide synthase, idole	Nidulanin A	75%
20	4.2	Type 1 polyketide synthase	Andrastin A	100%
20	4.6	Type 3 polyketide synthase, non-ribosomal peptide synthetase-like	Choline	100%
21	5.3	Terpene	PR-Toxin	100%
21	5.11	Type 1 polyketide synthase	Burnettiene A	87%

n.d.—the predicted secondary metabolite gene cluster has no descriptor associated to the entry.

## Data Availability

Genome assembly and annotations are available in the NCBI repository under bioproject PRJNA1097562 or through a Zenodo data repository (https://doi.org/10.5281/zenodo.10944763). Sequencing data will be deposited in the NCBI Short-read archive (SRA) prior to publication.
